# Hyperscanning fNIRS data analysis using multiregression dynamic models: an illustration in a violin duo

**DOI:** 10.3389/fncom.2023.1132160

**Published:** 2023-07-27

**Authors:** Diego Carvalho do Nascimento, José Roberto Santos da Silva, Anderson Ara, João Ricardo Sato, Lilia Costa

**Affiliations:** ^1^Departamento de Matemática, Facultad de Ingeniería, Universidad de Atacama, Copiapó, Chile; ^2^Department of Statistics, Federal University of Bahia, Salvador, Brazil; ^3^EcMetrics Pesquisa de Mercado, Salvador, Brazil; ^4^Departamento de Estatística, Universidade Federal do Parana, Curitiba, Brazil; ^5^Center of Mathematics, Computing and Cognition, Universidade Federal do ABC, São Bernardo do Campo, Brazil

**Keywords:** dynamic network, state-space models, causal inference, dual brain, interactive social neuroscience

## Abstract

**Introduction:**

Interpersonal neural synchronization (INS) demands a greater understanding of a brain's influence on others. Therefore, brain synchronization is an even more complex system than intrasubject brain connectivity and must be investigated. There is a need to develop novel methods for statistical inference in this context.

**Methods:**

In this study, motivated by the analysis of fNIRS hyperscanning data, which measure the activity of multiple brains simultaneously, we propose a two-step network estimation: Tabu search local method and global maximization in the selected subgroup [partial conditional directed acyclic graph (DAG) + multiregression dynamic model]. We illustrate this approach in a dataset of two individuals who are playing the violin together.

**Results:**

This study contributes new tools to the social neuroscience field, which may provide new perspectives about intersubject interactions. Our proposed approach estimates the best probabilistic network representation, in addition to providing access to the time-varying parameters, which may be helpful in understanding the brain-to-brain association of these two players.

**Discussion:**

The illustration of the violin duo highlights the time-evolving changes in the brain activation of an individual influencing the other one through a data-driven analysis. We confirmed that one player was leading the other given the ROI causal relation toward the other player.

## 1. Introduction

The brain is formed by a network in which different regions share information Horwitz (2003). This brain network can be studied through *functional connectivity*, which represents the patterns of statistical dependence on the activity of distinct brain regions, or through *effective connectivity*, which means the causal influences of the activity of one region over another. The variance-covariance matrix and the Bayesian network (BN) are examples of methods used to estimate functional connectivity. Other methods can be used to study effective connectivity, such as dynamic causal modeling (DCM) and the multiregression dynamic model (MDM). For a given directed network structure, the MDM models the data at each node as a linear combination of the parent nodes with time-varying connectivity parameters. According to Queen and Smith (1993), the MDM can distinguish between directed graphs corresponding to the same statistical dependence structure (which map onto the same undirected graphs), allowing for the accurate estimation of the directions of edges (a simple example of this is also discussed here). Moreover, the MDM can be observed as more than a static network (similar to BN). Alternative examples of these dynamic methods can be found in Burger et al. (2009), who used the dynamic Bayesian network (DBN) and hidden Markov models (HMM) used in human-robot interaction (DBN and HMM are a particular case of MDM).

In any case, the problem of finding a common pattern of brain connectivity for a given individual profile (healthy or with a specific disease, e.g., Alzheimer's disease) is not trivial owing to the presence of noise and the high-dimensionality of the data (Nascimento et al., 2020; Pinto-Orellana et al., 2020). For the MDM, Costa et al. (2015) presented a score-based learning network approach using a linear programming problem that finds the most likely network structure while considering the subset comparison through their maximum posterior probability (MAP) estimation. The authors demonstrated the usefulness of their method on functional Magnetic Ressonance Imaging (fMRI) data as it becomes unfeasible as the number of nodes (i.e., brain regions) increases.

In addition to this challenge, in the field of social neuroscience, understanding how the activity of the brain might influence the activity of another brain, which is known as brain-to-brain activity correlation, is also desirable. As examples, we considered a classroom where the teacher and the students interact or an orchestra where the musicians and the conductor interact. Konvalinka and Roepstorff (2012) describes how mutually interacting brains can be useful in social interaction. Balconi et al. (2017) studied the effects of strategic cooperation on intra- and inter-brain connectivity by functional near-infrared spectroscopy (fNIRS). Jiang et al. (2019) developed a study entitled “BrainNet: a multi-person brain-brain interface for direct collaboration between brains,” among others.

Hyperscanning studies—measuring the activity of multiple brains simultaneously—is a promising (flexible) paradigm regarding the measurement of brain activity from two or more people simultaneously while they are interacting. This could reveal interpersonal brain mechanisms underlying interaction-mediated brain-to-brain coupling Scholkmann et al. (2013). One experiment that could be conducted to this end, focusing on two brains' observations, is the study of violin duos playing together. The fNIRS could be used to overcome functional magnetic resonance imaging constraints, but few dynamic data-driven models have been proposed. Thus, we aimed to apply a dynamic graphical model to show dynamic changes in intersubject brain activity dependence over time.

### 1.1. Interaction-mediated brain-to-brain activity correlation

In recent decades, part of the neuroscience field has focused on demonstrating the nervous system and its function through individuals' behavior (and inter-relations) (Liu and Pelowski, 2014). For instance, some studies have discussed the brain connectivity structure by gender (Wang et al., 2009; Baker et al., 2016; Pan et al., 2017), age (Gong et al., 2009), or using other characteristics such as intelligence (Song et al., 2008; Van Den Heuvel M. et al., 2008; van den Heuvel M. P. et al., 2008), psychoactive ingestion (Palhano-Fontes et al., 2019), and meditative states (Brefczynski-Lewis et al., 2007; Brewer et al., 2011; Hasenkamp and Barsalou, 2012). Nevertheless, all of them have targeted different methodologies related to neuroanatomy. These methodologies also understand the brain connection patterns in human actions, such as opening and closing eyes (or moving any other body part), reading, writing, playing sports, learning, sleeping, creating memories, and recalling these memories (Hahn et al., 2018).

However interpersonal neural synchronization (INS) demands a greater understanding of the influence that a brain may carry on others rather than observing only a single brain response per time (for further details, see Babiloni and Astolfi, 2014). Hyperscanning studies are based on the simultaneous acquisition of brain dynamics during a cooperative task, as a joint action or decision-making (Liu et al., 2016, 2017).

Li et al. (2020) studied the cooperative behavior among basketball players, in which significant INS was observed due to the performed joint-drawing task but not the control task. Nguyen et al. (2020) investigated the neural processes related to transferring information across brains during naturalistic teaching and learning, underlying the effective communication of complex information across brains in classroom settings.

With more than only linking actions across subjects, studies have revealed that inter-individuals' neural representation can even build memories, thereby promoting brain integration at some influential level. Zadbood et al. (2017) uncovered the intimate correspondences between memory encoding and event construction and highlighted the essential role that our common language plays in the process of transmitting one's memories to other brains. Chen et al. (2017) elucidated that the neural patterns during perception are systematically altered across people into shared memory representations for real-life events.

Most methods used in hyperscanning fMRI and fNIRS studies are static or temporal correlation (Cui et al., 2012; Reindl et al., 2022; Balconi and Angioletti, 2023; Morgan et al., 2023; Wei et al., 2023) and Granger-based causality (Zhang et al., 2017; Chen et al., 2020, 2023; Pan et al., 2021; Zhao et al., 2022). Examples of the former method are the partial correlation coefficient and wavelet transform coherence (WTC). These methods are used to estimate functional connectivity and, therefore, do not distinguish the causal relationships between nodes. Nonetheless, according to neuroimaging literature, the latter is used to estimate directed functional connectivity (Bilek et al., 2022), and according to some studies, Granger causality theory cannot be suitable for hemodynamic data (Smith et al., 2011; Babiloni and Astolfi, 2014). Therefore, these approaches do not study putative causal synchrony between brains (Bilek et al., 2022). Thus, Bilek et al. (2022) used dynamic causal modeling (DCM) in the study of social interaction to estimate the causal effect one brain might have on another. However, DCM is a method for testing hypotheses, and initially specifying some candidate network structures is necessary.

This study uses the MDM with the Bayes factor (MDM-BF) that considers the contemporaneous relationship between regions, i.e., the nodes are related at the same time, in contrast, for example, to a DBN in which the past of the parents is connected with the present of the child. Moreover, the Kalman filtering method is used to estimate the effective connectivity in a simple way. However, in contrast to DCM, it can capture the dynamic nature of social interaction. A similar objective can be observed in Li et al. (2021) and Wang et al. (2022), in which the researchers used a data-driven approach based on sliding windows and *k*-mean clustering to capture the dynamic modulation of inter-brain synchrony patterns. However, it is based on temporal correlation and does not estimate effective connectivity.

The MDM appears to accommodate fMRI data well (see e.g., Costa et al., 2015); therefore, it has been used in this study for the first time with fNIRS data. Furthermore, this study proposes a new method that can be used to learn the directed acyclic graph (DAG) structure using the MDM faster than the method already available in the literature (MDM-IPA) because this method does not create the need to check all possible parents for each node. This can be especially useful in social neuroscience—which involves estimating both inter-brain and intra-brain connections and thus studying brain function on the subject and dyadic levels.

This novel method consists of two steps: in the first one, the tabu search algorithm would be applied to find a partial conditional directed acyclic graph (partial conditional DAG). The tabu search is a combinatorial optimization algorithm used to find an optimal network structure by local searches, as explained in the next section. Then, the Markov equivalence class would be found, that is, DAGs that encode the same statistical properties, and the DAG with the highest log predictive likelihood (LPL) score from the MDM would be chosen. This search method can also be used to estimate individual brain networks.

Based on such evidence, which highlights the possibility of studying the brain-to-brain activity correlation, in the next subsection, we have discussed an extension class of DBNs that can be used to represent these brain dynamic and causal structures (from now on, whenever we refer to causality, it is associated with effective connectivity via MDM-BF, unless indicated differently).

This study is divided into four parts. In Section 2, we have described the fNIRS data analyzed and the methods used to estimate brain connectivity as a graph-based model. Section 3 describes the evaluation, through synthetic data, of the robustness of the dynamic graphical model. Then, Section 4 presents the empirical results, and finally, Section 5 presents the discussion of the proposed method and the findings.

## 2. Materials and methods

We present a proof-of-concept based on a hyperscanning experiment in which the human interaction is investigated from brain-to-brain activity dependence. The methodological approach adopted in this study was divided into four main steps, aiming to estimate the brain's dynamics and interactions. The developed R script in this study is available at https://github.com/ProfNascimento/MDM-BF (accessed on April 20th, 2023).

### 2.1. The data

This study dataset was first presented as a case study experiment (Balardin et al., 2017) that considered two individuals who played in a violin duo. In the current study, we have investigated the brain-to-brain coupling (and the direction) and explored which brain regions of a violinist are linked to the other.

**Figure 1:**
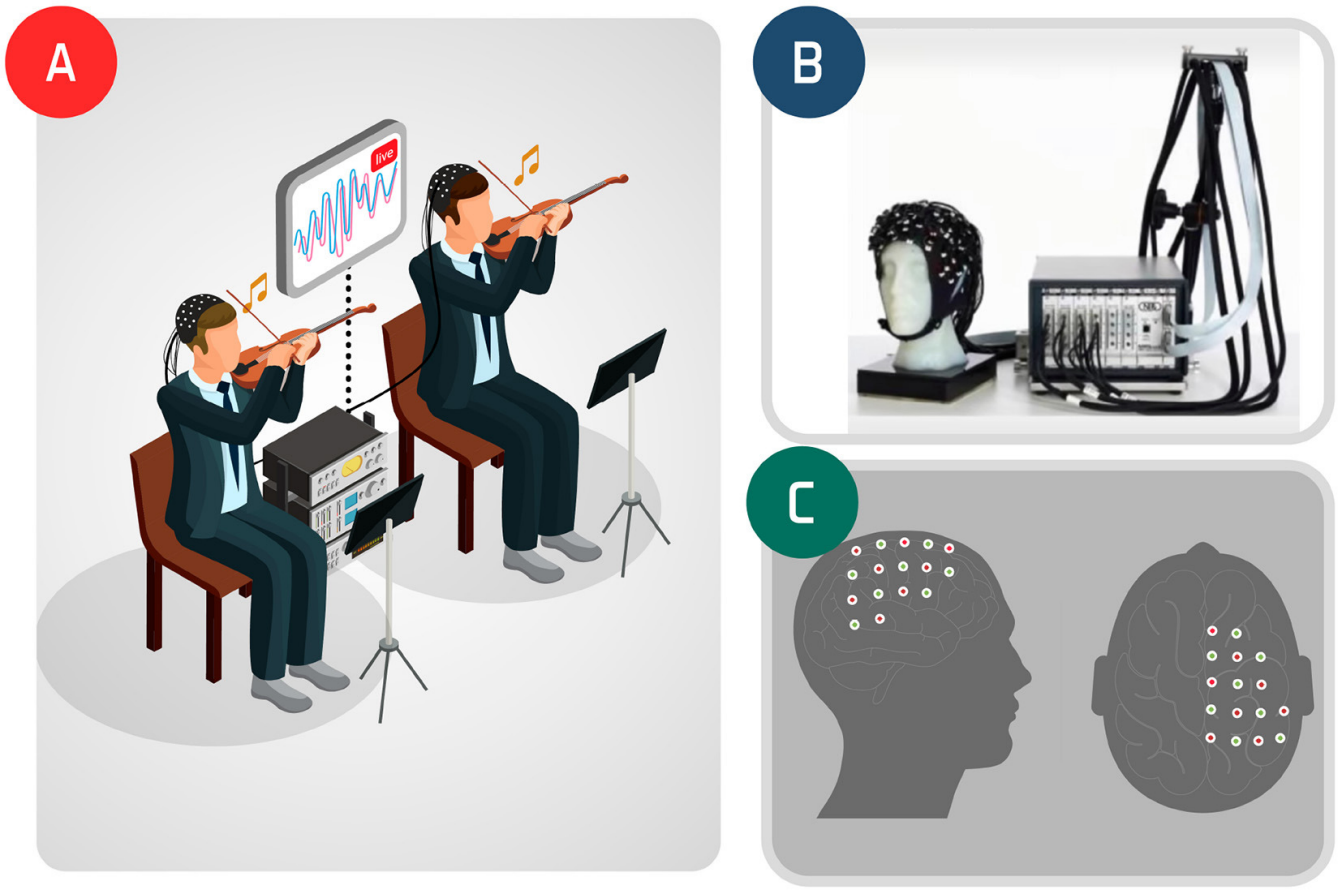
Violin duo experiment: inter-subjects' experiment icon **(A)**; fNIRS **(B)** and the observed brain region **(C)**.

The fNIRS signals acquired are demonstrated in Figure 1 (for further experiment details, see Balardin et al., 2017). Hemodynamic changes were obtained from the optical changes collected using the continuous wave functional near-infrared spectroscopy system (NIRScout 16x16, NIRx Medical Technologies, Glen Head, NY) with 16 LED light sources (760 and 850 nm) and 16 detectors per musician, at a sampling rate of 7.81 Hz. Channel aggregation was conducted by considering the EEG 10-10 system in which the optodes were placed.

The participants were at a professional level, right-handed, and men aged 41 and 50 years old. They were instructed to play a 32-s stretch of Allegro, by Antonio Vivaldi, from Concerto No 1 in E major, op. 8, RV 269, “Spring." Hyperscanning was performed considering 23 channels of the right motor hemisphere and the temporoparietal junction of the two violinists (Balardin et al., 2017). The first 36 s of acquisition refers to the duo playing and the remaining refers to a resting-state condition.

### 2.2. Dataflow

**Figure 2:**
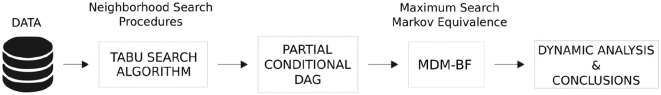
Visual summary of the methodological framework. Tabu search algorithm reduced the sub-graph structure to be sought. Then, the result was transformed into a partial conditional DAG, and the outcome was compared using the MDM-BF.

Figure 2 demonstrates a data processing flow chart. Given the computational cost of searching for the likely topology of the graph, at first, the tabu search algorithm was applied using Bayesian networks to reduce the sub-graph structure to be sought. Then, the result was transformed into a partial conditional directed acyclic graph (DAG), enabling it to proceed under the causal inference paradigm (Pearl, 2009; Oates et al., 2015). After that, the most likely undirected network structure found was implied in Markov equivalent graphs, and then, the MDM was applied to unravel directionality through the maximization of the LPL, that is, Bayes factor. By adopting a particle filter supposing Gaussian noises (often known as the Kalman filter), we compared the MAP graphs to obtain the most likely DAG. Once the DAG is defined, the MDM-BF can present the dynamic strength of these estimated links. This adopted methodology enables the estimation of complex brain structures whenever the number of vertices (nodes) is >11 with a sample size > 100 points (for further details, see Costa et al., 2015, Table 01, p. 456), without computational constraints.

### 2.3. The multiregression dynamic model

The MDM models multivariate time series, studying putative causal relations among its variables over time (Queen and Smith, 1993; Queen and Albers, 2009). This class of models is extremely powerful, given that it can discriminate complex multivariate relations up to a finite r-th time series, with length *t*, set as (*Y*_*t*_(1), *Y*_*t*_(2), ..., *Y*_*t*_(*r*)). Moreover, the joint distribution (*P*(*Y*_*t*_(1), *Y*_*t*_(2), ..., *Y*_*t*_(*r*))) is estimated regardless of the presence of a Gaussianity (for further details, please see Queen and Smith, 1993, who have retained the proof of the consistency of this method of non-Gaussian processes). The MDM is formed by using univariate regression dynamic linear models (DLMs), in which the observation *Y*_*t*_(*r*) is regressed onto its parents, with Gaussian residuals, such as in Equation (1).


(1) $$\begin{array}{l} Y_{t}(r)\sim N(F_{t}(r{)}^{\prime }\theta_{t}(r),V_{t}(r))\;\\ \;\theta_{t}\sim N(G_{t}\theta_{t-1},W_{t}), \end{array}$$

where *Y*_*t*_(*r*) is an observable variable at time *t* and brain region *r*, *r* = 1, …, *n* regions, *t* = 1, …, *T* time points, N denotes the Gaussian distribution, $\theta_{t}^{\prime }=\left(\theta_{t}{\left(1\right)}^{\prime },\ldots ,\theta_{t}{\left(n\right)}^{\prime }\right)$, $\theta_{t}{\left(r\right)}^{\prime }$ is the *p*_*r*_-dimensional parameter vector for *Y*_*t*_(*r*), and, when it is not intercepted, it represents the effective connectivity between node *r* and its descendent (also called parents). **F**_*t*_(*r*) is the set of the parents, and for nodes that do not have parents, *F*_*t*_(*r*) = 1. **G**_*t*_ increments the state equation in the form, giving extra variance.

In addition, **W**_*t*_(*r*) are *p*_*r*_ square matrices that form **W**_*t*_ = blockdiag{**W**_*t*_(1), …, **W**_*t*_(*n*)}. Note that, when **W**_*t*_(*r*) is a matrix with all elements equal to zero, the MDM becomes the BN. The parameters can be estimated using well-known Kalman filter recurrences over time (see, for example, West and Harrison, 2006). By so doing, the DLM is described by the set {**F**_*t*_(*r*), *Vt*, **G**_*t*_, **W**_*t*_}, although, in practice, establishing the **W**_*t*_ is challenging; therefore, a strategy called “discounting” (stochastic shifting) is adopted.


(2) $$\begin{array}{l} {\text{W}}_{t}=\frac{1-\delta }{\delta }\times C_{t-1}, \end{array}$$

where **W**_*t*_ is specified directly through a discount factor δ∈(0, 1], and ***C***_*t*−1_ is the posterior variance of **θ**_*t*_.

Before proceeding, three terminologies are important for distinguishing estimation processes: (*i*) Filtering is a procedure that aims to update the current estimates as new data are observed, i.e., ℙ(θ_*t*_∣*Y*_1:*t*_); (*ii*) smoothing is a retrospective analysis that has all the observations and calculates the conditional distribution θ given the heading from the complete data, ℙ(θ_*t*_∣*Y*_1:*T*_); and (*iii*) prediction is a forecast procedure that estimates the next observation based on the distribution, ℙ(θ_*t*+1_∣*Y*_1:*t*_).

### 2.4. The proposed learning network

The learning network process used in this study is 2-fold: (i) an estimation process of a Bayesian network structure, the tabu search algorithm, and (ii) choosing a structure via the MDM in Markov equivalent networks, that is, partial conditional DAG → MDM-BF. This methodological combination is an alternative to reduce the np-hard (dimensional) complexity search problem of the network estimation.

First, the initial estimation process is related to traditional methods in Bayesian networks (time-invariant structure). This approach was performed using a score-based method via standard tabu search with Bayesian information criterion (BIC). In general, this method searches for a Bayesian network structure that maximizes BIC. A Tabu search (Glover, 1986) may be viewed as a meta-heuristic algorithm to perform a greedy search and to avoid local minima. Thus, the procedure records information about changes recently made in BN structures, using one or more tabu lists. The tabu lists are managed by recording moves in a sequential order. Each time a new link is added to the end of a list, the oldest arc on the list is dropped from the beginning. Thus, each structure generated by adding or removing links is appraised by the BIC scoring (Nagarajan et al., 2013). The tabu algorithm adopted here can be found in the bnlearn package from the R software (R Core Team, 2022). Furthermore, every statistical analysis used in this study adopted the software R.

As it is well known that the BN search approaches have trouble distinguishing Markov equivalent structures, the next step was to find the graphs that are Markov equivalent to one resulting from the tabu search. Afterward, the network structure with the largest score of MDM among these Markov equivalent graphs was chosen. The pcalg package was used to obtain the partial conditional DAG.

Once the partial DAG structure was established, only a few subsets of possibilities remain to be sought. At this point, the maximum likelihood approach was adopted to determine the best options for the subgroup. The assumption from the MDM is that the standardized conditional one-step forecast errors have an approximate Gaussian distribution, although not based on stationary time series, and are serially independent with constant variance. Under these assumptions, the joint log predictive likelihood (LPL) has the closed form of a noncentral *t* distribution and is easily found in the Kalman filter (Costa et al., 2015). Remembering that *Y* = {*Y*_*t*_(1), ⋯ , *Y*_*t*_(*r*)} if time-invariant *Y* = {*Y*(1), ⋯ , *Y*(*r*)}, considering a multivariate non-central t distribution function


(3) $$\begin{array}{l} f(\text{𝕐}|\mu ,\sigma^{2}\text{Σ},\nu )=\frac{\text{Γ}[(\nu +r)/2]}{{\left(\pi \nu \right)}^{r/2}|\sigma^{2}\text{Σ}{|}^{1/2}\text{Γ}[\nu /2]}\\ {\left(1+\frac{\left(\text{𝕐}-\mu {)}^{\prime }{\text{Σ}}^{-1}(\text{𝕐}-\mu \right)}{\sigma^{2}\nu }\right)}^{-(\nu +r)/2} \end{array}$$

in which μ is the vector of the means and Σ is the variance-covariance matrix under the Bayesian framework


(4) $$\begin{array}{l} \mathbb{P}\left(\tau ,\text{𝕐}|\mu ,\sigma^{2}\text{Σ},\nu \right)\propto \mathbb{P}\left(\text{𝕐}|\tau ,\mu ,\sigma^{2}\text{Σ}\right)\mathbb{P}\left(\tau |\nu \right) \end{array}$$

(5) $$\begin{array}{l} \text{𝕐}|\tau ,\mu ,\sigma^{2}\text{Σ}\sim N\left(\mu ,\left(\sigma^{2}\text{Σ}/\tau \right)\right) \end{array}$$

(6) $$\begin{array}{l} \tau |\nu \sim Ga\left(\nu /2,\nu /2\right) \end{array}$$

and then assuming that the conditional distribution of each *Y*_*t*_(*r*) is given by the previous information set $F_{t-1}$, one can simply consider a regression structure for the conditional mean $\mu_{t}={\text{F}}_{t}{\left(r\right)}^{\prime }\theta_{t}\left(r\right)$ and Σ_*t*_ = *V*_*t*_


(7) $$\begin{array}{l} log\left(f\left(Y_{t}\left(1\right),\cdots \;,Y_{t}\left(r\right)|F_{t-1}\right)\right)=log\left(L\left({\text{F}}_{t}{\left(r\right)}^{\prime }\theta_{t}\left(r\right),V_{t}|F_{t-1}\right)\right)\\ =LPL\left({\text{F}}_{t}{\left(r\right)}^{\prime }\theta_{t}\left(r\right),V_{t}|F_{t-1}\right) \end{array}$$

Therefore, the LPL is the score of the MDM used in the learning network process, and in the following section, Section 3, a simple example of the ability of this score to distinguish two Markov equivalent graphs is given. It must be mentioned that local Gaussian models do not imply, necessarily, a posterior symmetrical multivariate distribution (for further details, see Queen and Smith, 1993).

## 3. Simulating the MDM

**Figure 3:**
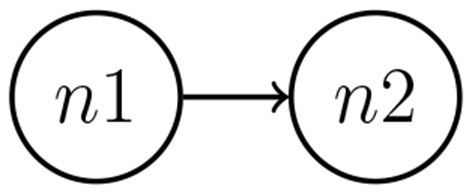
Data were simulated considering two nodes. The MDM method through Kalman filter estimation performs an estimate of direction and its time-varying strength.

This simulation study aimed to present the performance of the MDM in estimating the network structure and relationship strength (parameter θ) between the two nodes over time. Each time series contains 300 observations, that is, *t* = {1, …, 300}. Figure 3 represents the theoretical (known) network, in which node 1 (n1) is the parent of node 2 (n2). The data simulation was performed by using R software.

For instance, let us suppose that two signals, *n*1(*t*) and *n*2(*t*), are related, and they can be written as a first-order linear-Gaussian state-space model (these models presenting Gaussian noises are often called the Kalman filter, which is a special case of a particle filter for contemporaneous influence), as demonstrated in the following equations:


(8) $$\begin{array}{lll} n1_{t} & = & \theta_{t}^{\left(1\right)}+v_{t}^{\left(1\right)}\text{,}v_{t}^{\left(1\right)}\sim N\left(0,0.1^{2}\right) \end{array}$$

(9) $$\begin{array}{lll} n2_{t} & = & \theta_{t}^{\left(2\right)}+\theta_{t}^{\left(3\right)}n1_{t}+v_{t}^{\left(2\right)}\text{,}v_{t}^{\left(2\right)}\sim N\left(0,0.1^{2}\right), \end{array}$$

(10) $$\begin{array}{lll} \theta_{t}^{\left(k\right)} & = & \theta_{t-1}^{\left(k\right)}+w_{t}^{\left(k\right)}\text{,}w_{t}^{\left(k\right)}\sim N\left(0,0.1^{2}\right) \end{array}$$

in which *k* = {1, 2, 3}, and *v*^(1)^, *v*^(2)^, and *w*,(*k*) are independent. There are structural equations containing time-varying parameters $\theta_{t}^{\left(1\right)}$, $\theta_{t}^{\left(2\right)}$, and $\theta_{t}^{\left(3\right)}$. The parameters $\theta_{t}^{\left(1\right)}$ and $\theta_{t}^{\left(2\right)}$ are the drift that translates the strength for each node *i* at time *t*. The parameter $\theta_{t}^{\left(3\right)}$ is assumed to represent the form of the exchangeable sample information, in which (n1) impacts into (n2), and then, later, this is observed as a causal strength (in neuroscience, the effective neuronal connectivity).

**Figure 4:**
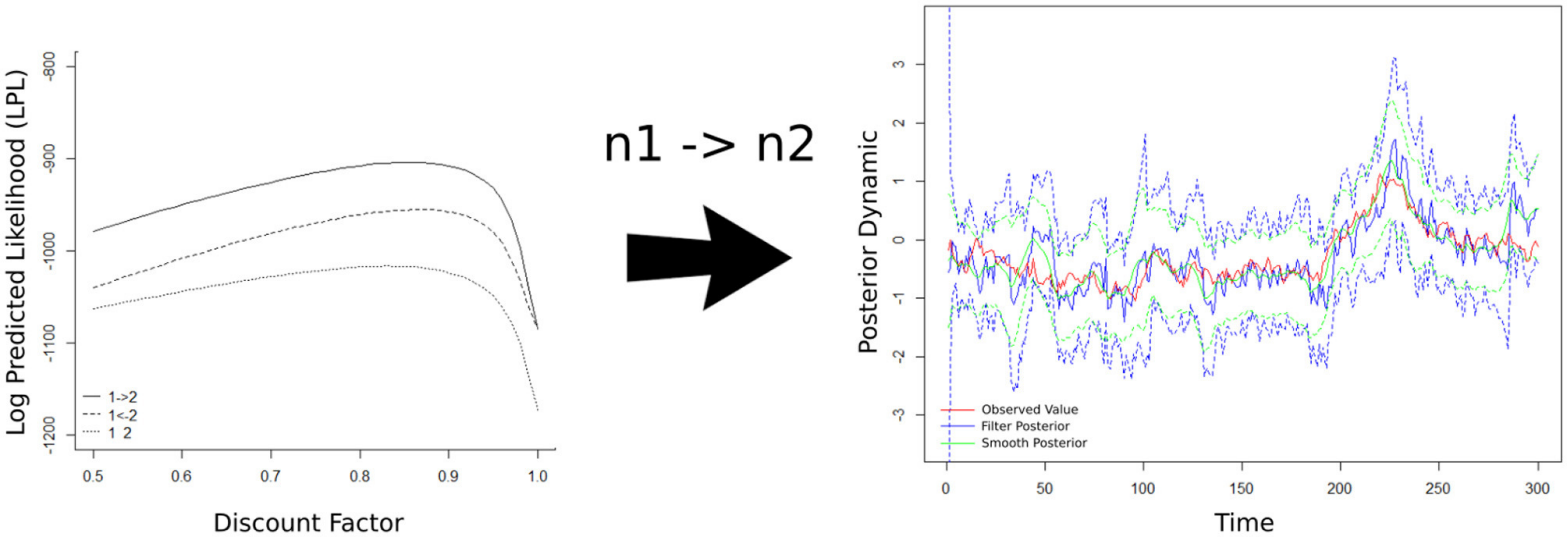
The result of the data-driven model MDM-BF is represented in two parts, *structure selection* and *dynamic estimation*. The left-hand panel describes the score (LPL) of each possible network arrangement (as a time-varying score). Three possible network outcomes were adjusted, n1 impacting n2 (solid line), n2 impacting n1 (dash line), and both nodes being independent (dot line), and at all times, n1 → n2 presents a higher score (then, the most likely structure). The right-hand panel shows the dynamic of the link strength, given the selected network (n1 → n2), that is, the dynamic of $\theta_{t}^{\left(3\right)}$. The red line represents the true value, the blue solid line is the smooth posterior mean (and the dashed lines are the credible intervals containing 95%), and the green line is the filter posterior mean.

After generating and processing the synthetic network, the left-hand panel of Figure 4 shows the estimated LPL for each possible network set (that is, n1 → n2, n2 → n1, and both independent nodes) by a discount factor (DF). In the inference process of the MDM-BF, ${\text{W}}_{t}^{\left(r\right)}$ can be written in the function of a DF that represents the loss of information in the change of parameter **θ** between times *t*−1 and *t*. The DF varies between zero and one, in a way that the closer the DF is to one, the more stable the system is. When DF assumes the value one, ${\text{W}}_{t}^{\left(r\right)}$ is the matrix of zeros, and the MDM becomes a BN (Costa et al., 2015). After selecting the most likely model, the strength dynamism of the connection is calculated through a time-varying parameter (θ_*t*_) approach. The right-hand panel of Figure 4 shows the true value and the MDM dynamic estimation regarding the causal effect between the nodes.

The steps are summarized in Algorithm 1 summarized as follows:

In this case, the network with the highest LPL values was network n1 → n2, which generated the data. Markov equivalent networks have the same dependency relations between the nodes and have equivalent/equal LPL. Therefore, when the discount factor is 1, as we mentioned, there is no variation in the state parameters over time, and the MDM simply becomes a BN. Then, unsurprisingly, the direction n1 → n2 or n2 → n1 does not matter (see the left-hand panel of Figure 4). Thus, this study presents an indication that the MDM-BF is efficient in distinguishing structures that can be Markov equivalent. Here, we described the simplest case of a network structure (with only two nodes) for the sake of simplicity and visualization; nevertheless, the results are expandable to higher complexities (see e.g., in Costa et al., 2015). The next section discusses the results obtained in neuroscience application tasks.

**Algorithm 1:** Causal inference MDM-BF schematic (based on Figure 2).

Read the DATA

Apply the TABU search using the Bayesian Network to estimate the invariant structure

if *n*1↛*n*2 or *n*2↛*n*1 **then**
*n*1, *n*2 are independent
else
*n*1 → *n*2 or *n*2 → *n*1
Then, n1 is connected to n2 but partial conditional, that is, the direction will be ignored at this point. For a greater number of nodes, every combination will be tested.
end **if**

Calculate LPL from the TABU search subgroup, the partial conditional DAG (*n*1 → *n*2 or *n*2 → *n*1).

Then, the choice will be the DAG with the maximum LPL (that is, the directions that are established).

Once the DAG is set, the dynamic linear model is adjusted on the DAG regression structure (time-varying parameters estimation step, that is MDM-BF).

## 4. Experimental results

**Figure 5:**
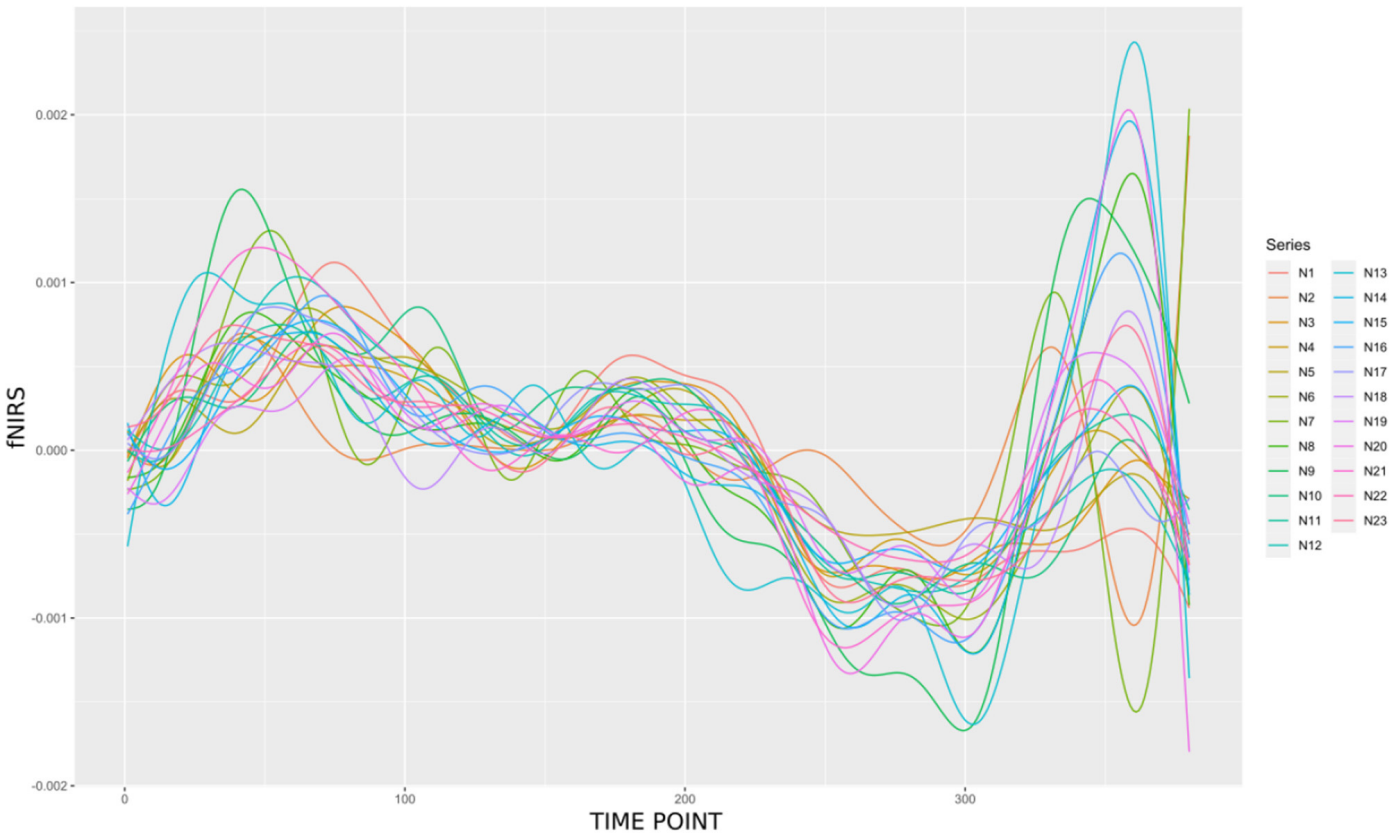
A butterfly plot illustrating the 23 oxyhemoglobin (HbO) signals from violinist A.

fNIRS enables simultaneous recording, making it possible to study the influence of brain-to-brain coupling through social interaction experiments. Figure 5 shows the fNIRS data during the music duration (218 time points) from violinist A in the 23 channels.

### 4.1. Dynamic brain-to-brain evolution

**Figure 6:**
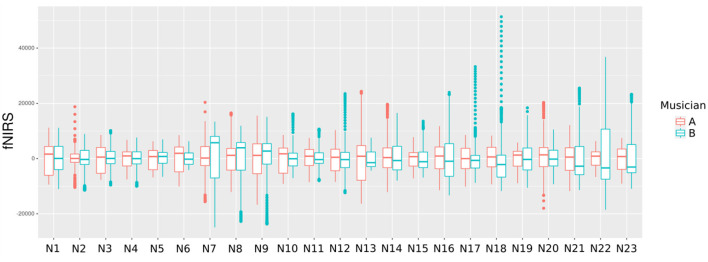
A boxplot comparing the 23 HbO signals among both musicians. This graph depicts the dispersion of the HbO of each violinist.

The study of the network involved the brains of the two violinists and considered 46 nodes, the first 23 ones corresponding to the first subject, and the remaining ones to the second subject (Figure 6). The learning of the network structure was carried out by comparing the Markov equivalent networks to the graph estimated by the tabu method and using the LPL of the MDM. The combination of the tabu search algorithm, partial conditional DAG, and the MDM-BF helped enhance the computation efficiency, bringing back the best network structure chosen for each subject.

The brain activation dynamic was analyzed by using the state-space model, obtained from the MDM-BF through its posterior mean smoothing process. It is worth mentioning that only positive connections (ignoring the few small negative estimates, as physiological interpretations are difficult to make) were presented, which enabled us to take into account their neurological interpretability. Moreover, these connections represent the neural activation resulting from one region's influences over another.

**Figure 7:**
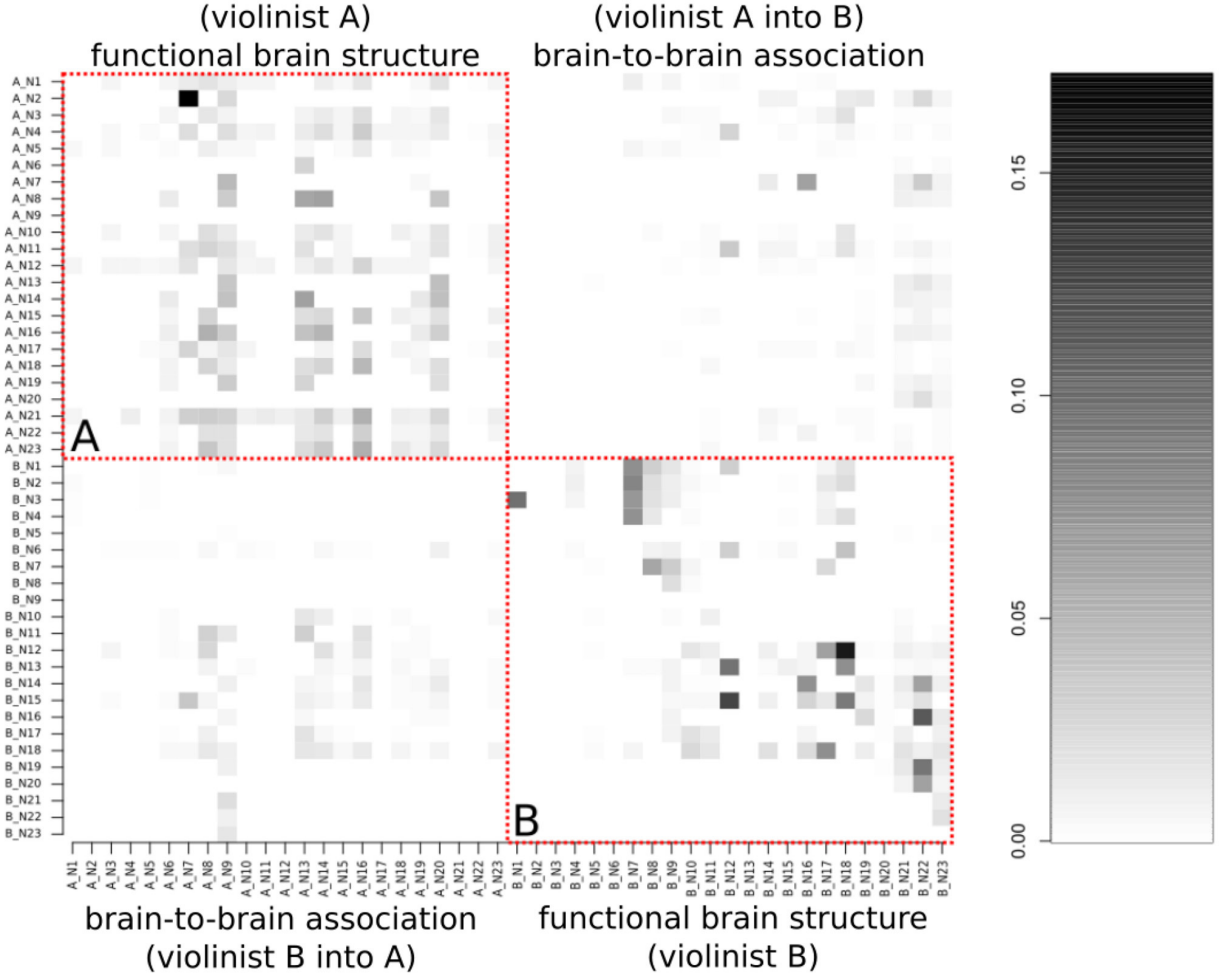
The average of the posterior mean of the strength of connectivity *i*→*j* over time, in which *i* indexes rows and *j* columns (representing each observed HbO). This matrix is divided into four blocks, in which the diagonal block is related to the musicians' activation of their own brain (in the red dashed line), and the anti-diagonal block to the brain-to-brain activity correlations. **(A)** (top-left quadrant) is from the functional brain structure of violinist A, and **(B)** (bottom-right quadrant) is from violinist B.

Figure 7 presents the results of the graph-based MDM, as a matrix in which each element is the average of the posterior mean of the strength of connectivity *i*→*j* over time, in which *i* (parents) indexes rows and *j* (children) indexes columns (the matrix causal relation direction is described from the row to the column). Moreover, this matrix is divided into four blocks, in which the diagonal block is intrasubject connectivity, for violinist A, at the top left square and for violinist B, at the lower right square. In contrast, the antidiagonal block shows intersubject connectivity, in which the influence of the brain regions of violinist A to B is at the top right, whereas the influence of violinist B to A is at the lower left.

**Figure 8:**
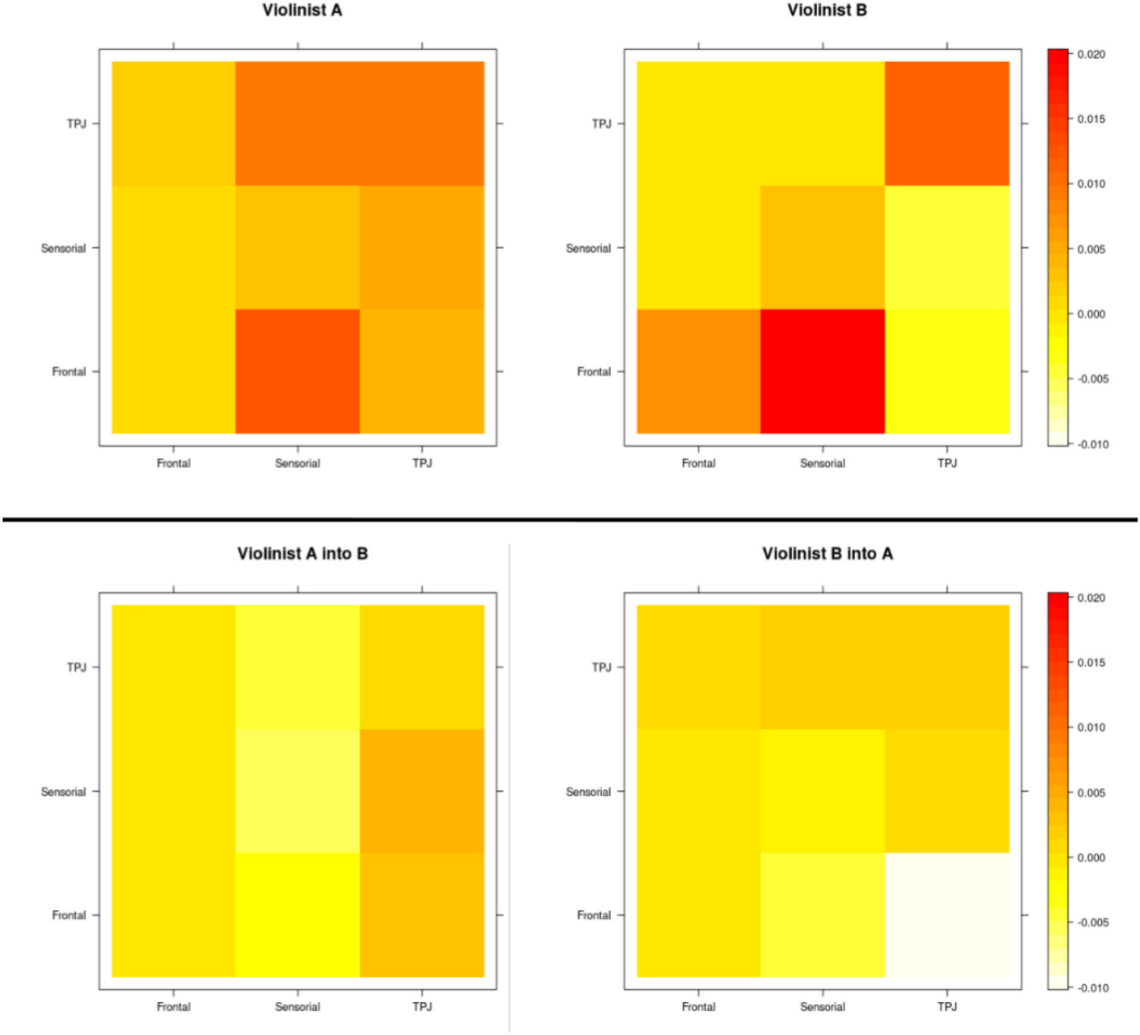
ROIs' mean representation, according to the 10-10 EEG montage, of the intra-individuals connectivity as well as the inter-individual based on the MDM-BF.

Stronger connections are represented in the matrix by the darker color, while lighter regions represent weak or absent connections. As expected, the strongest connections are in the primarily diagonal block, which represents the intrasubject brain connections. The anti-diagonal block reveals that the intersubject connectivities are less prevalent and less strong. Thus, based on a standard 10-10 EEG montage, we aggregated the channel numbers 1, 2, 3, and 4 as dorsal frontal Regions of Interest (ROIs) 6, 7, 8, 10, and 11 as sensorimotor ROIs, and 12, 13, 15, 16, and 21 as temporoparietal junction (TPJ) and calculated the mean of the influence of each region. A summary of the intra-individual connectivity (summed through the ROIs' mean according to the 10-10 EEG montage) vs. inter-individual connectivity is represented in Figure 8. The causal direction is from the row to the column, that is, the highest ROI activity from violinist A was from the frontal into the sensorimotor, whereas for violinist B, the causal relation from TPJ into sensorimotor was not strong. Moreover, the strongest observed values across inter-brains were from all three ROIs from violinist A to the TPJ from violinist B (left-bottom picture in the third column).

Broadly speaking, these causal relationships are estimated based on the best-adjusted joint probability distribution between the NIRScout (16 LED light sources leading to 23 time series from each violinist) represented as a network. In the best model, first, the partial DAGs obtained can be said to present the intra- and inter-individual connections, and then, the conditional independence of the time series is incorporated according to the assumptions of the model. First, the best network structure for each participant is estimated independently, and then, the hyperscanning network structure is also estimated independently from the others. Nonetheless, the three network dynamics cannot be regarded as totally independent because only thetas can show that (if they are zeros). Moreover, a “partializing relationship” can be observed across structures conditioned to the inter-individual vs. intra-individual as the obtained DAGs.

**Figure 9:**
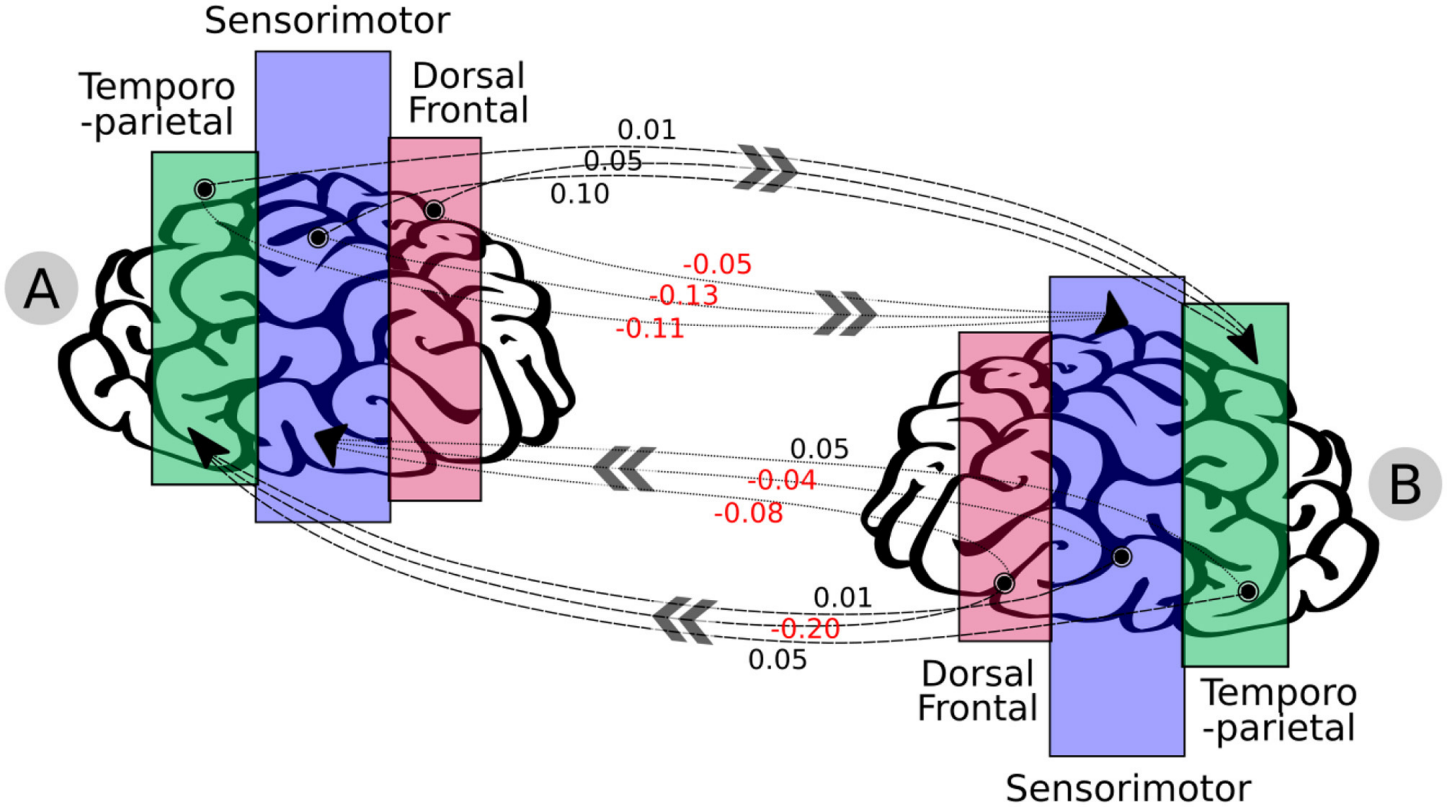
Graph-based representation resulting from the musicians' brain-to-brain synchrony through the ROIs: dorsal frontal, sensorimotor, and temporoparietal. Moreover, this graph is the summation of channels that represent the mutual interactions across the musicians' brains (estimated from the MDM's antidiagonal blocks demonstrated in Figure 7). High activity was seen to influence the sensorimotor and temporoparietal for both musicians; nonetheless, no effective correlation of brain-to-brain activity was observed directly toward the dorsal frontal. The numbers in black are positive values, and those in red are negative values. The results highlight that musician B followed musician A, as the highest positive value goes from sensorimotor (musician A) → TPJ (musician B), and the highest negative value goes from dorsal frontal (musician B) → TPJ (musician A), suggesting a reverse causal direction.

Figure 9 shows the visual representation of the summation of this antidiagonal block as a graph. For instance, the most influenced regions were sensorimotor and TPJ, as results of the INS, and the results demonstrated that violinist B was influenced by violinist A, as the highest positive value goes from the sensorimotor (violinist A) → TPJ (musician B), and the highest negative value goes from dorsal frontal (violinist B) → TPJ (musician A), suggesting a reverse causal direction.

The uncertainty can be associated with confidence intervals (CI) toward this ROI causal connectivity, which was obtained through the non-parametric bootstrap algorithm (Carpenter and Bithell, 2000), using the MDM average of each posterior. We used the nptest package while considering the mean statistic method, a confidence level of 0.95, and the number of replicates of 50,000 (Table 1). A statistical significance was observed from the dorsal frontal channels' behavior (musician A) → TPJ (musician B), sensorimotor channels' behavior (musician A) → sensorimotor (musician B). In the other direction, it was observed from the dorsal frontal channels' behavior (musician B) → sensorimotor (musician A), dorsal frontal (musician B) → TPJ (musician A), and TPJ (musician B) → TPJ (musician A). The other relations were not statistically significant.

**Table 1:** Non-parametric bootstrap of the ROIs' mean.

**Regions influence**	**CI 95%**	
Frontal_A → TPJ_B	0.00001	0.00581
Sensor_A → Sensor_B	−0.01053	−0.00107
Frontal_B → Sensor_A	−0.00790	−0.00161
Frontal_B → TPJ_A	−0.02136	−0.00311
TPJ_B → TPJ_A	0.00080	0.00422

**Figure 10:**
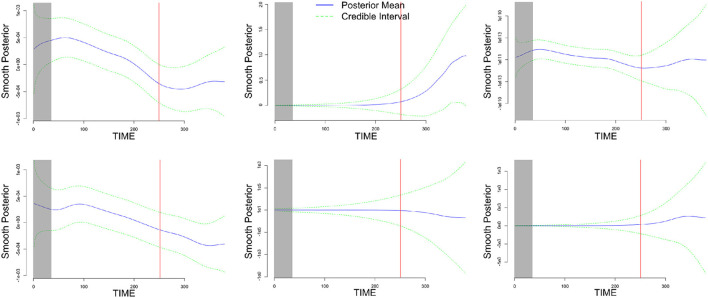
An illustration of the dynamics of the six parameters related to the INS inter-activated fNIRS, through their smoothing posterior mean time-varying parameters (solid blue line), with 95% credible interval (dashed green line). Thus, the resting-stage moment is represented by the rectangular-shaded areas and the red-solid line represents the time-point of the end of the music. Based on the two higher causal ROI relations (in module), the three top charts are associated with some sensorimotor channels' behavior (musician A) → TPJ (musician B), and the three bottom charts are associated with some dorsal frontal channels' behavior (musician B) → TPJ (musician A).

Additionally, by using the MDM class, one can make inferences regarding the time-varying strength of the network's links. For instance, the dynamic change among some channels was noticeable, especially during the resting-stage period (delimited by after the red line), as shown in Figure 10. It is clear that the estimated dynamic of the network links was captured by the MDM.

It is clear that intra-subject effective connectivity is stronger than the brain-to-brain coupling strength. Tasks involving music were reported previously and appear to induce brain activation (Li et al., 2015). Berkowitz and Ansari (2010) discussed the importance of the observed brain region (right TPJ, also called rTPJ) in musicians. Luo et al. (2014) showed neuroimaging toward long-term musical training, which shows an impact on emotional and cognitive function, suggesting the presence of neuroplasticity in the rTPJ.

The sensorimotor and TPJ ROIs presented a greater activation influence from the INS; furthermore, the MDM could capture that musician B was following musician A, which also provides some evidence toward the brain synchronization theory. The hypotheses for the ROIs' inter-individual connections relate to a distinct activation, for instance, highlighted in the literature as resulting from the assessment of different body movements (Kimura, 1977) or even emotions felt through visual stimuli due to the execution of the activity (Zaitchik et al., 2010).

## 5. Final remarks

The current study proposes the MDM-BF for fNIRS data obtained in hyperscanning experiments, i.e., simultaneous acquisition, while two or more subjects are interacting. The illustration in a violin duo confirmed the existence of influences of one brain over the other. In the individual brain network analysis for each violinist, it was observed that, although the brain regions work together, some areas play different roles. In other words, some regions connect to others with greater strength. Moreover, this data-driven analysis demonstrated, through their INS estimation, that the influence between violinists is not symmetric and also time-evolving. Therefore, the MDM-BF appears to be a competitive model that is better for hyperscanning studies (due to estimating the effective connectivity) than other methods based on correlation or the consideration of static connections (which only estimate functional connectivity), corroborating similar results that have already been presented in other fields of neuroscience (Costa et al., 2015). In addition, the MDM-BF estimated the inter-brain network using the contemporaneous relationship between regions, without needing to consider the Granger causality.

In the INS analysis [also known as Thinking Through Other Minds (TTOM)], the regions activated on the violinists are represented by the ROI activation and, through the data-driven model, corresponded to the expected results observed in the experimentation (in which musician A was the leader in the duo); that is, the quantification obtained from the MDM-BF brain region connections are highlighted, as shown in Figure 9. However, as this study considered only a pair of violinists, further studies targeting the brain mapping should be conducted to associate the pattern with more in-depth details regarding those connections. In general, the connections estimated by the MDM-BF for the joint matrix of connections represent the brain ROIs' activity correlations and their dynamic over time, in which all regions present positive meaning and strong connections.

Different for DCM, in this study, social brain network structures could be better explored. The study also analyzed the synchronized dynamical system = globally, as well as the communication of specific parts of the brain. Moreover, the novel procedure for the learning structure network that is presented in this study, or others that are used with the MDM (as the MDM-BF or the MDM-DGM) can be easily applied in other scenarios, such as communication and computer-mediated cooperation games. Furthermore, this approach can be suitable for other neuroscience studies that aim to estimate brain networks and have a large number of nodes. A natural next step will be to incorporate informative priors, in which targets transform the researchers' prior knowledge into hyper-parameters. In addition, parametric space shrinkage should be investigated as an alternative to score-based structure selection. In other words, as a complement to the MDM-BF method, the number of time-varying parameter estimations can be reduced based on some a priori information or some specific criteria.

## Data availability statement

The original contributions presented in the study are included in the article/supplementary material, further inquiries can be directed to the corresponding author.

## Author contributions

All authors listed have made a substantial, direct, and intellectual contribution to the work and approved it for publication.
